# Estimating Children’s Soil/Dust Ingestion Rates through Retrospective Analyses of Blood Lead Biomonitoring from the Bunker Hill Superfund Site in Idaho

**DOI:** 10.1289/ehp.1510144

**Published:** 2016-01-08

**Authors:** Ian von Lindern, Susan Spalinger, Marc L. Stifelman, Lindsay Wichers Stanek, Casey Bartrem

**Affiliations:** 1 TerraGraphics Environmental Engineering, Inc., Moscow, Idaho, USA; 2 U.S. Environmental Protection Agency (EPA), Region 10, Seattle, Washington, USA; 3 U.S. EPA, Office of Research and Development, Research Triangle Park, North Carolina, USA

## Abstract

**Background:**

Soil/dust ingestion rates are important variables in assessing children’s health risks in contaminated environments. Current estimates are based largely on soil tracer methodology, which is limited by analytical uncertainty, small sample size, and short study duration.

**Objectives:**

The objective was to estimate site-specific soil/dust ingestion rates through reevaluation of the lead absorption dose–response relationship using new bioavailability data from the Bunker Hill Mining and Metallurgical Complex Superfund Site (BHSS) in Idaho, USA.

**Methods:**

The U.S. Environmental Protection Agency (EPA) in vitro bioavailability methodology was applied to archived BHSS soil and dust samples. Using age-specific biokinetic slope factors, we related bioavailable lead from these sources to children’s blood lead levels (BLLs) monitored during cleanup from 1988 through 2002. Quantitative regression analyses and exposure assessment guidance were used to develop candidate soil/dust source partition scenarios estimating lead intake, allowing estimation of age-specific soil/dust ingestion rates. These ingestion rate and bioavailability estimates were simultaneously applied to the U.S. EPA Integrated Exposure Uptake Biokinetic Model for Lead in Children to determine those combinations best approximating observed BLLs.

**Results:**

Absolute soil and house dust bioavailability averaged 33% (SD ± 4%) and 28% (SD ± 6%), respectively. Estimated BHSS age-specific soil/dust ingestion rates are 86–94 mg/day for 6-month- to 2-year-old children and 51–67 mg/day for 2- to 9-year-old children.

**Conclusions:**

Soil/dust ingestion rate estimates for 1- to 9-year-old children at the BHSS are lower than those commonly used in human health risk assessment. A substantial component of children’s exposure comes from sources beyond the immediate home environment.

**Citation:**

von Lindern I, Spalinger S, Stifelman ML, Stanek LW, Bartrem C. 2016. Estimating children’s soil/dust ingestion rates through retrospective analyses of blood lead biomonitoring from the Bunker Hill Superfund Site in Idaho. Environ Health Perspect 124:1462–1470; http://dx.doi.org/10.1289/ehp.1510144

## Introduction

### Ingestion Rate Background

Consumption of fine soil and dust particulates, especially by young children, is the dominant route of exposure for lead and other contaminants (Laidlaw et al. 2014; Landrigan et al. 1975; Lanphear et al. 1998, 2003). Childhood soil and dust ingestion occurs via multiple pathways, including hand-to-mouth transfer, mouthing of objects, and contaminated food. These pathways are dependent on individual behaviors, exposure time, and environmental conditions (Zahran et al. 2013a). Accurate estimates of the soil and household dust ingestion rate (IR) pathway are needed to assess children’s exposures and health risks associated with trace metals and persistent organic chemical residues in the home or play environment, and to make informed cleanup decisions.

Early estimates of soil/dust IRs in children were based on studies of trace elements in soil and feces, yielding uncertain estimates due to analytical uncertainty, limited sample size, and short study duration (Batelle 2005; Doyle et al. 2010; Sedman and Mahmood 1994; Stanek et al. 2012; U.S. EPA 2011, 2012). Currently, national U.S. Environmental Protection Agency (EPA) central tendency soil/dust IRs of 60 mg/day (children 6 weeks to < 12 months of age) and 100 mg/day (children 1 to < 6 years of age) are based on these tracer studies (U.S. EPA 2011). More recent studies have used dermal transfer to estimate soil and dust IRs. Ozkaynak et al. (2011) modeled the frequency of hand and object mouthing in children 3 years to < 6 years of age, resulting in a mean total soil/dust IR of 68 mg/day (95th percentile: 224 mg/day). Similarly, Wilson et al. (2013) used a mechanistic model including parameters for particle loading on skin, transfer to hands, hand surface area, mouthing surface area, hand-to-mouth frequency, saliva dissolution, and exposure time, to estimate an average combined soil/dust IR of 61 mg/day for children 7 months to 4 years of age. Meta-analysis of four major studies using stochastic modeling of the most reliable tracers resulted in an average soil ingestion estimate of 26 mg/day (95th percentile: 79 mg/day) for children 1–8 years of age (Stanek et al. 2012). Findings from large-scale reviews and integration of data from tracer, mechanistic, validation modeling/measurement, and empirical relation (biomonitoring/environmental concentration) studies suggest that mean IRs in children are < 100 mg/day and may be as low as 40–80 mg/day (Bierkens et al. 2011; Moya and Phillips 2014).

Soil/dust IR and bioavailability are sensitive parameters in the U.S. EPA Integrated Exposure Uptake Biokinetic (IEUBK) Model for Lead in Children. The IEUBK model currently uses default IRs ranging from 85 to 135 mg/day for 6-month- to 6-year-old children and 30% absolute bioavailability for ingested soil and indoor dust (U.S. EPA 2013). The first use of the IEUBK model to develop site-specific cleanup levels was at the Bunker Hill Mining and Metallurgical Complex Superfund Site (BHSS) in northern Idaho (CH2M Hill 1991; TerraGraphics 1990; U.S. EPA and IDHW 1991, 1992). The dose–response relationship observed between soil, dust, and blood lead levels (BLLs) was consistently lower at the BHSS than IEUBK model predictions using the default parameters (TerraGraphics 1990; von Lindern et al. 2003b). This was nominally attributed to lower soil/dust bioavailability (18%), although it was acknowledged that the reduced dose response was likely a combination of lower bioavailability and IRs (von Lindern et al. 2003b).

### BHSS Background

In 1974, soon after the lead smelter operators bypassed emission controls destroyed by a baghouse fire, > 95% of children 1–9 years of age living within 3 mi of the smelter had BLLs exceeding 40 μg/dL (Yankel et al. 1977). Lead health interventions have been ongoing since that time. The smelter closed in 1981 and remediation began in 1986, representing one of the world’s largest, most comprehensive, and well-documented lead health response cleanups (U.S. EPA 2005, 2010; von Lindern et al. 2003a, 2003b). From 1988 through 2002, soil from > 3,500 properties within the 21-mi^2^ area surrounding the smelter was removed and replaced with up to 1 ft of clean fill averaging ≤ 50 mg/kg. Hundreds of families with children received lead health education and in-home follow-up investigations through a local Lead Health Intervention Program (LHIP). The LHIP tested children’s BLLs, achieving participation rates > 50% among 0- to 9-year-old children for 15 consecutive years through door-to-door recruitment and incentive payments. Annual blood lead survey results were used to prioritize soil cleanup until the Remedial Action Objective (RAO) of < 5% of children with BLLs ≥ 10 μg/dL was achieved. From 1988 through 2002, homes of young children (0–6 years), pregnant women, and older children with BLLs ≥ 10 μg/dL were remediated first, regardless of location within the site. Beginning in 1994, all soils in contiguous neighborhoods with lead levels ≥ 1,000 mg/kg were removed and replaced, regardless of BLLs. This cleanup prioritization coupled with families moving within the affected communities resulted in a dynamic, complex combination of soil/dust exposures affecting individual children.

Blood lead data, collected during the seasonal peak in late summer, were matched to dust lead concentrations (from samples collected from household vacuum cleaners) and soil lead data to monitor the relationship between children’s BLLs and environmental exposures to ensure cleanup was effective. Four variables were used to quantify soil and dust exposures throughout the cleanup: house dust, yard soil, neighborhood soil, and community soil lead concentrations. The neighborhood soil variable is the mean of all yard soils within a specific radius of the home, excluding the home’s yard soil lead concentration. This was calculated for 200-ft, 500-ft, and 1,000-ft radii. The community soil variable is the mean of all yard soils within the community, excluding the home and neighborhood radius soil lead concentrations. The prioritized cleanup rapidly reduced the number of children residing in homes with soil lead concentrations ≥ 1,000 mg/kg and markedly decreased yard soil exposures for those families. Neighborhood soil lead concentrations progressively declined until the block-by-block cleanup strategy was implemented in 1994, and then decreased faster as contiguous neighborhoods were remediated. Community soil lead mean concentrations declined steadily until 2002 when yard soil replacement was mostly complete. House dust lead exposures (dust lead concentrations from homes of children with BLL measurements) decreased following the yard, neighborhood, community, and industrial complex cleanups but lagged the community soil means by a decade or more (von Lindern et al. 2003a).

By 2002, children’s mean BLLs decreased to 2.2 μg/dL. In 2013, the health district conducted the first comprehensive blood lead survey since 2002, recruiting an estimated 50% of children 6 months to 9 years of age living within the 21-mi^2^ area using incentive payments and door-to-door solicitation. The geometric mean BLL among 1- to 5-year-old children tested was 2.2 μg/dL (SD ± 1.8) compared with the most recent U.S. mean of 1.3 μg/dL (CDC 2013), with 2 of 276 children having levels ≥ 10 μg/dL, indicating that the cleanup continues to meet the RAO of 95% of children < 10 μg/dL (TerraGraphics 2015). Over the 15 years of active cleanup (1988–2002), education, and intervention, the LHIP amassed approximately 5,400 blood lead observations (referred to as the parent database) from nearly 2,340 individuals, yielding 2,176 records of blood/soil/dust lead concentrations (TerraGraphics 2004; von Lindern et al. 2003b, 2003a).

Subsequent to the cleanup at the BHSS, the U.S. EPA adopted an *in vitro* methodology to estimate site-specific bioavailability of lead in soil and dust (U.S. EPA 2012). This methodology was applied to a subset of archived soil and dust samples from the BHSS, and results were applied to the parent database. The objective of this study was to estimate age-specific soil/dust IRs through reanalysis of the dose–response relationship using new soil and house dust lead bioavailability data. In light of uncertainties and limitations of fecal tracer soil ingestion studies, these site-specific estimates likely have broader application to the IEUBK model and to human health risk assessment.

## Methods

Blood lead samples collected from children participating in the LHIP were obtained through written informed consent from parents as well as child assent. The annual LHIP surveys are public health actions undertaken by state and local health authorities. TerraGraphics secured approval from the University of Idaho’s Institutional Review Board for this project. No additional survey data or samples were collected from human subjects for this analysis.

### Sample Analyses

In total, 271 samples (193 house dust samples, 73 yard soil samples, and 5 quality control samples) sieved to 80 mesh (or < 0.177 mm) were analyzed for total lead (Method 6010B) and *in vitro* bioaccessibility (TerraGraphics 2012; U.S. EPA 2007, 2012). U.S. EPA’s *in vitro* assay measures the solubility, or bioaccessibility, of lead in soil and dust samples to estimate (*in vivo*) bioavailability. The 80 mesh sieve for both soil and dust was initiated at the BHSS in 1974 and focuses analyses on particle sizes more likely to adhere to hands and other surfaces and be ingested by children (Panhandle District Health Department et al. 1986; Ruby and Lowney 2012). Archived soil and dust samples collected between 1986 and 2002 were retrieved from storage. Those with intact seals, legible identification numbers, and sufficient mass for analysis were then checked to ensure that blood lead data and information on child age and sex, home location, and property remediation status were available. A temporal and geographic subset of samples meeting these criteria was randomly selected and analyzed at the laboratory. Reanalyzed soil and dust lead concentrations were compared to historical values using linear regression. *In vitro* bioaccessibility results were converted to *in vivo* relative bioavailability and absolute bioavailability (ABS) following U.S. EPA methods using comparison to a lead acetate reference (0.5) following Equation 1 (U.S. EPA 2007, 2009, 2012):

ABS = (0.878 × IVBA – 0.028) × 0.5, [1]

where IVBA = *in vitro* bioaccessibility.

Community mean ABS values for unremediated yard soils and house dust, and site-wide ABS means for postremediation soils were integrated into the parent database. Annual site-wide ABS means were calculated using a weighted average of bioavailable lead (product of concentration and bioavailability) from remediated and unremediated yards.

### Quantitative Analyses

Soil and dust partitions, age-specific IRs, and lead uptake from sources other than soil and dust were determined through structural equations modeling (SEM). SEM is a statistical multivariate methodology appropriate for pathways analysis, defined as a network of linear relations between variables. SEM was applied by von Lindern et al. (2003b) to reflect the exposure pathways of yard, community, and neighborhood soils (Ullman and Bentlar 2003). The 2003 SEM was repeated using absorbed and bioavailable lead (instead of blood and total soil and dust lead levels), using SAS® software version 8 (SAS Institute Inc.). Several combinations of variables, including neighborhood soil means using radii of 200 ft, 500 ft, and 1,000 ft and age- and year-specific soil and dust categorical variables (i.e., grouped by both age and calendar year), were alternatively added, and model fit was evaluated by five criteria: *a*) convergence, *b*) chi-square probability test (*p* > 0.05), *c*) goodness of fit index (GFI) (> 0.90), *d*) parameters with significant t-statistics (*p* < 0.05), and *e*) parameter performance in subsequent IEUBK model analyses, described below (Carey 1998; SAS Institute Inc. 2008; Suhr 2006; Wothke 2010). Both the chi-square and GFI measure the difference between the expected and observed covariance matrices. Higher chi-square probability indicates better fit. The GFI ranges from 0 to 1.0, with higher values indicating better fit (Jöreskog and Sörbom 1988; SAS Institute Inc. 2008). SEM equations were solved using mean values for the independent variables and model parameters to estimate: *a*) soil and dust lead pathway parameters, *b*) neighborhood and community soil effects on lead uptake, *c*) age-specific and temporal effects in lead intake and uptake, and *d*) source partition scenarios for use in subsequent IEUBK modeling.

### Ingestion Rate Estimates

Total lead uptake (μg/day) was calculated by dividing the measured BLLs (μg/dL) by the age-specific biokinetic slope factors, referred to as CR^–1^ (day/dL), used in the original IEUBK model (Harley and Kneip 1985; Jacobs Engineering et al. 1989; Kneip et al. 1983; TerraGraphics 1990, 2012; U.S. EPA 1994). Total lead uptake was partitioned into components used in the IEUBK model: air, diet, water, and soil/dust. Lead uptake from soil and dust was estimated by partitioned dust, yard soil, neighborhood soil (used only in the SEM), and community soil subcomponents by subtracting air, dietary, and drinking-water uptakes estimated from the IEUBK model default values (U.S. EPA 2001), as shown in Equation 2:

*UP_sd_* = [(*C_d_* × *IR_d_* × *ABS_d_*) + (*C_ys_* × *IR_ys_* × *ABS_ys_*) + (*C_cs_* × *IR_cs_* × *ABS_cs_*) + (*C_ns_* × *IR_ns_* × *ABS_ns_*)] = *UP_tot_ –* [*UP_air_* + *UP_diet_* + *UP_water_*], [2]

where *UP* = lead uptake (μg/day); *C* = concentration (mg/kg); IR = ingestion rate (mg/day); *ABS* = absolute bioavailability (unitless); and (subscripts): *sd* = combined soil/dust sources; *d* = house dust; *ys* = yard soil; *cs* = community soil; *ns* = neighborhood soil (SEM); *tot* = total sources; *air* = airborne source; *diet* = dietary source; *water* = water source.

Equation 2 can be rearranged to calculate total soil/dust IRs [*IR_sd_* (mg/day) is the sum of *IR_d_*, *IR_ys_*, *IR_cs_*, and *IR_ns_*] by assigning partition coefficients, i.e., fractional contributions to total soil/dust ingestion by each source, as follows in Equation 3:

*IR_sd_* = 1,000 × {*UP_sd_* / [(*C_d_* × *PT_d_* × *ABS_d_*) + (*C_ys_* × *PT_ys_* × *ABS_ys_*) + (*C_cs_* × *PT_cs_* × *ABS_cs_*) + (*C_ns_* × *PT_ns_* × *ABS_ns_*)]}, [3]

where *PT* = partition coefficient.

Partition coefficients used in these analyses included the IEUBK model default, those originally developed to support BHSS cleanup criteria, and values derived from SEM. Partition coefficients, resulting age-specific soil/dust IRs (using Equation 3), and bioavailability were input to the IEUBK model batch-mode analyses (IEUBKwin v1.1 build 11) to compare predicted and observed BLLs. The combined IR and partition scenarios showing best-predicted BLLs were evaluated by linear regression and sums of squared error (SSE). The slope nearest to 1.0 coupled with the highest *r^2^*, highest *F*-statistic, and lowest sum of squared residuals from linear regression, as well as the SSE (squared difference between observed and predicted geometric mean BLLs), were used to determine the scenario(s) that best represent observed BLLs. The age-specific soil and dust IR estimates were then determined based on these scenario(s).

## Results

### Sample Analysis

The selected subset of historical data was considered generally representative of the parent database (e.g., lead concentration and child’s age) (Table 1). The reanalyzed soil and dust lead concentrations were not significantly different from historical results (*r^2^* = 0.99, *p* < 0.01, *n* = 73; and *r^2^* = 0.91, *p* < 0.01, *n* = 193, respectively), indicating that samples were not compromised during storage. The reanalyzed sample results are summarized in Table 2. Mean soil bioavailability ranged from 30% to 39% by community, averaging 33% (SD ± 4%); dust bioavailability ranged from 27% to 30%, averaging 28% (SD ± 6%). Three “clean” soil samples were obtained in 2011 from borrow piles used to replace contaminated property soils. No clean yard soil samples were previously collected and archived. Consequently, these three samples represent postremediation replacement clean soils, and bioavailability results averaged 15% (SD ± 0.6%; data not shown). Linear regression relating soil and dust bioavailability to lead concentration showed a weak relationship (*r^2^* = 0.15, *p* = 0.0006 and *r^2^ =* 0.045, *p* = 0.0028, respectively).

**Table 1:** Comparison of the parent BHSS database with the subset of records selected for reanalysis (historical data).

City	Parent data set**						Selected subset					
	Minimum	Maximum	Average	SD	Geometric mean	Geometric SD	Minimum	Maximum	Average	SD	Geometric mean	Geometric SD
Kellogg	Parent data set* n* *=* 3,054						Selected subset *n *= 118					
Age (years)	0	9	5.1	2.7	—	—	1	9	5.5	2.6	—	—
Blood lead (μg/dL)	1	54	6.4	4.7	5.1	2.0	2	41	7.6	5.7	6.3	1.8
Soil lead (mg/kg)	100	13,400	954	1,625	274	4.4	100	6,930	1,407	1,849	435	5.2
Dust lead (mg/kg)	32	52,700	1,213	2,839	733	2.4	88	5,530	1,373	1,093	985	2.3
Page	Parent data set* n* = 161						Selected subset *n *= 15					
Age (years)	0	9	5.1	2.6	—	—	1	9	4.3	2.8	—	—
Blood lead (μg/dL)	1	26	7.0	4.7	5.7	1.9	3	12	5.6	2.4	5.2	1.5
Soil lead (mg/kg)	53	3,480	557	668	287	3.2	100	1,670	541	420	387	2.5
Dust lead (mg/kg)	69	2,070	678	496	478	2.6	86	1,680	706	567	467	2.9
Pinehurst	Parent data set* n* = 1,369						Selected subset *n *= 117					
Age (years)	0	9	5.1	2.6	—	—	1	9	5.2	2.4	—	—
Blood lead (μg/dL)	1	26	4.6	3.1	3.8	1.9	1	17	4.3	2.6	3.7	1.7
Soil lead (mg/kg)	31	3,060	438	424	312	2.3	37	1,700	469	356	369	2.0
Dust lead (mg/kg)	22	15,000	639	1,053	417	2.4	45	15,000	625	1,427	383	2.3
Smelterville	Parent data set* n* = 642						Selected subset *n *= 57					
Age (years)	0	9	4.9	2.7	—	—	1	9	4.5	2.6	—	—
Blood lead (μg/dL)	1	55	7.0	5.4	5.6	2.0	2	30	7.5	4.9	6.4	1.7
Soil lead (mg/kg)	100	10,700	953	1,921	245	4.3	100	8,170	1,037	1,821	242	4.8
Dust lead (mg/kg)	54	11,300	1,127	1,257	757	2.5	393	4,210	1,387	807	1,190	1.8
Wardner	Parent data set* n* = 173						Selected subset *n *= 5					
Age (years)	0	9	5.2	2.7	—	—	1	8	4.8	3.1	—	—
Blood lead (μg/dL)	1	20	6.6	3.8	5.5	1.9	2	8	4.6	2.2	4.2	1.6
Soil lead (mg/kg)	100	34,800	759	2,925	224	3.5	100	13,200	3,104	5,705	484	9.6
Dust lead (mg/kg)	130	6,000	1,005	1,112	700	2.3	307	2,220	1,147	697	959	2.1

**Table 2:** Community averages of reanalyzed archived soil and house dust samples.

City	Soil			Dust		
	*n*	Soil lead (mg/kg) (mean ± SD)	Soil ABS (%) (mean ± SD)	*n*	Dust lead (mean ± SD) (mg/kg)	Dust ABS (%) (mean ± SD)
Kellogg	24	2,656 ± 1,624	34 ± 3	66	1,179 ± 934	28 ± 6
Page	7	778 ± 417	33 ± 4	12	753 ± 529	27 ± 5
Pinehurst	33	569 ± 463	32 ± 4	75	762 ± 2,131	28 ± 6
Smelterville	8	4,136 ± 2,192	39 ± 2	36	1,239 ± 550	30 ± 4
Wardner	1	2,030	30	4	892 ± 415	27 ± 5
Overall	73	1,686 ± 1,748	33 ± 4	193	996 ± 1,472	28 ± 6
ABS, absolute bioavailability.						

### SEM Analyses

Several plausible SEM combinations met the model acceptance criteria. In each accepted model, bioavailable lead in dust and soils from the home yard, neighborhood, and community were all significant independent predictors of total blood lead uptake. Based on experience with the BHSS cleanup and development of the parent database, numerous combinations of spatial, temporal, and age-specific variable constructs and database time periods were explored (data not shown). Of the three neighborhood radii, 500 ft showed the best fit by combined chi-square test and parameter *t*-values. Age-specific coefficients for dust concentration among the youngest children (6 to < 24 months old) were significant (*p* < 0.01), implying different IRs, with a significant intercept representing uptake from other sources. Coefficients for age-specific and year-specific soil concentration variables were not significant (*p* > 0.05). The SEM with temporal variables showed marginally significant (*p* = 0.05) positive dust coefficients for 6- to 23-month-old children in 1994–1998, suggesting higher dust IRs during those years.

Source partitions using three SEM combinations were evaluated in subsequent IEUBK model analyses: Model 1 (1989–2002 database) included a term allowing calculation of year-specific IRs, and model 2 (1989–1998 database) and model 3 (1989–2002 database) assumed constant source contributions and IRs throughout each respective time period (Tables 3 and 4). Soil/dust IRs and source partitions were estimated by substitution of mean soil and dust lead concentrations in the model equations.

**Table 3:** Structural equations modeling (SEM) results.

Variables	Model 1 (1989–2002)			Model 2 (1989–1998)			Model 3 (1989–2002)		
	Slope coefficient	*t*-Value^*a*^	Standardized coefficient	Slope coefficient	*t*-Value^*a*^	Standardized coefficient	Slope coefficient	*t*-Value^*a*^	Standardized coefficient
UP_tot _(Equation 4)
ln(UP_d_)	0.1347	8.43	0.2575	0.1466	7.95	0.2762	0.1360	8.50	0.2598
ln(DUSTage0–1)	0.0450	2.80	0.0132	0.0440	2.24	0.0116	0.0450	2.79	0.0132
ln(DUSTage1–2)	0.0501	4.06	0.0273	0.0613	6.23	0.0333	0.0667	7.45	0.0363
ln(DUST1994–1998)	0.0336	1.95	0.0128	—	—	—	—	—	—
ln(UP_ys_)	0.0611	6.09	0.1027	0.0516	4.82	0.0866	0.0601	5.99	0.1010
ln(UP_ns_)	0.0647	3.30	0.1364	0.0661	2.41	0.1396	0.0636	3.24	0.1341
ln(UP_cs_)	0.1594	6.03	0.3439	0.0954	2.75	0.2050	0.1571	5.94	0.3389
Intercept	0.3639	3.34	0.1316	0.7666	5.55	0.2670	0.3820	3.52	0.1382
Error	—	—	0.2098	—	—	0.2021	—	—	0.2100
Bioavailable dust lead (Equation 5)
ln(UP_ys_)	0.1039	7.57	0.0914	0.1054	7.31	0.0938	0.1039	7.57	0.0914
ln(UP_ns_)	0.0751	2.77	0.0828	0.1126	3.01	0.1262	0.0751	2.77	0.0828
ln(UP_cs_)	0.3350	9.35	0.3782	0.2582	5.50	0.2944	0.3350	9.35	0.3782
Intercept	2.3390	16.52	0.4418	2.5994	14.67	0.4804	2.3339	16.52	0.4418
Error	—	—	0.1523	—	—	0.1468	—	—	0.1523
Baseline bioavailable lead (μg/dL)	1.4			2.2			1.5
Baseline bioavailable dust lead (mg/kg)	37.0			48.1			36.9
*n*	2,034			1,571			2,034		
Goodness of fit index	0.9995			0.9999			0.9998		
χ^2^	4.7284			0.598			1.5347		
Degrees of freedom	3			2			2		
Pr > χ^2^	0.1928			0.7416			0.4642		
*r*^2^ Total uptake	0.9560			0.9591			0.9559		
*r*^2^ Bioavailable dust lead	0.9768			0.9785			0.9768		
Abbreviations: χ^2^, chi-square; cs, community soil; d, dust; DUSTage0–1, bioavailable dust lead if the child was 6–11 months; DUSTage1–2, bioavailable dust lead if the child was 12–23 months; DUST1994–1998, bioavailable dust lead if the year was 1994, 1995, 1996, 1997, or 1998. ln, natural log; ns, neighborhood soil; Pr, probability; *r*^2^, *r*-squared; tot, total; UP, uptake; ys, yard soil. ^***a***^*t*-Values ≥ 1.96 are equivalent to *p*-values < 0.05.									

**Table 4:** Structural equations modeling (SEM) results for soil/dust contributions (%).

Variables	Model 1 (1989–2002)			Model 2 (1989–1998)			Model 3 (1989–2002)		
	0–2 years	2–9 years	Value^*a*^	0–2 years	2–9 years	Value^*a*^	0–2 years	2–9 years	Value^*a*^
Contribution of dust/soil ingestion
House dust	40	37	40	48	45	50	41	38	40
Yard	30	30	30	28	30	25	30	31	30
Neighborhood	11	11	10	9	10	10	11	11	10
Community	19	23	20	15	15	15	18	20	20
Contribution to lead in blood
House dust			17			22			16
Yard			35			34			33
Neighborhood			14			15			14
Community			34			29			37
^***a***^Values are rounded to total 100%.									

Model 2, shown in Equations 4 and 5 (chi-square test: *p* = 0.7416, *n* = 1,571; Table 3), was selected based on performance in subsequent IEUBK modeling:

ln(*UP_tot_*) = [0.1466 × ln(*C_d_* × *ABS_d_*)] + [0.0516 × ln(*C_ys_* × *ABS_ys_*)] + [0.0440 × ln(*C_d_* × *ABS_d_* × age0–1)] + [0.0613 × ln(*C_d_* × *ABS_d_* × age1–2)] + [0.0661 × ln(*C_ns_* × *ABS_cs_*)] + [0.0954 × ln(*C_cs_* × *ABS_cs_*)] + 0.7666 [4]

ln(*C_d_* × *ABS_d_*) = [0.1054 × ln(*C_ys_* × *ABS_ys_*)] + [0.1126 × ln(*C_ns_* × *ABC_cs_*)] + [0.2582 × ln(*C_cs_* × *ABS_cs_*)] + 2.5994, [5]

where ln = natural log; *C_ns_* = neighborhood soil arithmetic mean using 500-ft radius (mg/kg); age0–1 = 1 for 6–11 months, otherwise 0; age1–2 = 1 for 12–23 months, otherwise 0; *ABS_cs_* applies to both *C_ns_* and *C_cs_* values.

The SEM standardized regression coefficients (Table 3) yielded partition coefficients of 50% house dust/25% yard soil/10% arithmetic mean neighborhood soil/15% arithmetic mean community soil (50/25/10/15) (Table 4) used in subsequent calculation of age-specific IRs.

### Ingestion Rate Estimates

**Figure 1:**
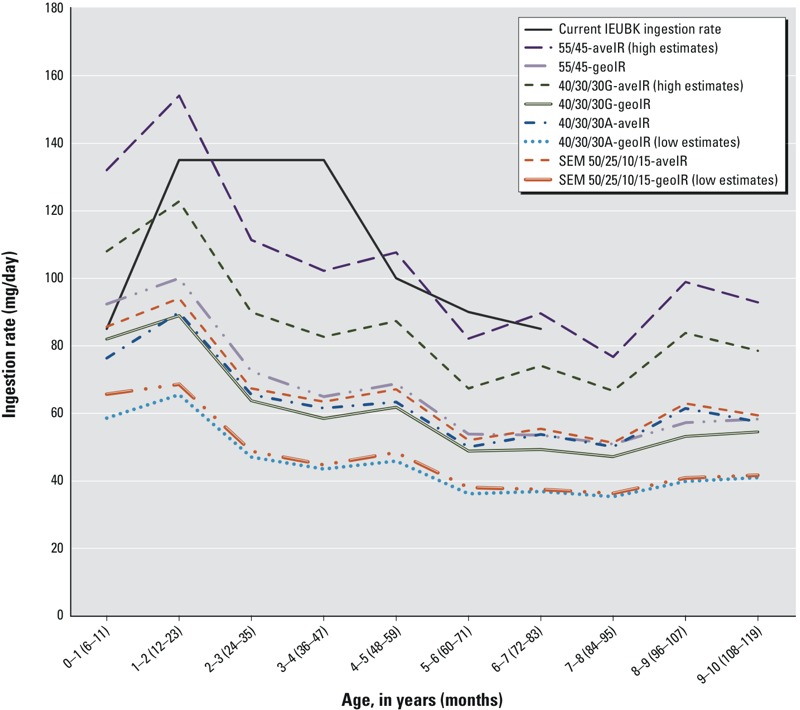
Arithmetic and geometric mean age-specific soil/dust ingestion rates (IRs) for four soil/dust partition scenarios. Included are current Integrated Exposure Uptake Biokinetic (IEUBK) model IRs and calculated age-specific mean soil/dust IRs for the four partition scenarios. For each age (6 months–9 years), arithmetic mean IRs (aveIR) and geometric mean IRs (geoIR) are shown. 55/45 is the partition of dust/yard soil, 40/30/30 is the partition of dust/yard/community soil, and SEM 50/25/10/15 is the partition of dust/yard/neighborhood/community soil. Corresponding numeric data, with 95% CI and percentile distributions for each model and age, are provided in Table S1.

Figure 1 summarizes arithmetic and geometric mean soil/dust IRs calculated for four source partition scenarios: *a*) the IEUBK model default 55% dust/45% yard soil (55/45), *b*) the original BHSS model applying 40% dust/30% yard soil/30% geometric mean community soil (40/30/30G) (Panhandle District Health Department 1986), *c*) the same partition using arithmetic average community soil (40/30/30A), and *d*) the SEM (50/25/10/15). Calculated IRs were observed in three general ranges. The highest IR estimates were arithmetic means for the 55/45 partition and are near the IEUBK model recommended values (also shown in Figure 1). Mid- and low-range IR estimates are approximately one-third and one-half lower, respectively [corresponding numeric data with 95% confidence interval (CI) and percentiles are provided in Table S1].

### IEUBK Model Results

**Figure 2:**
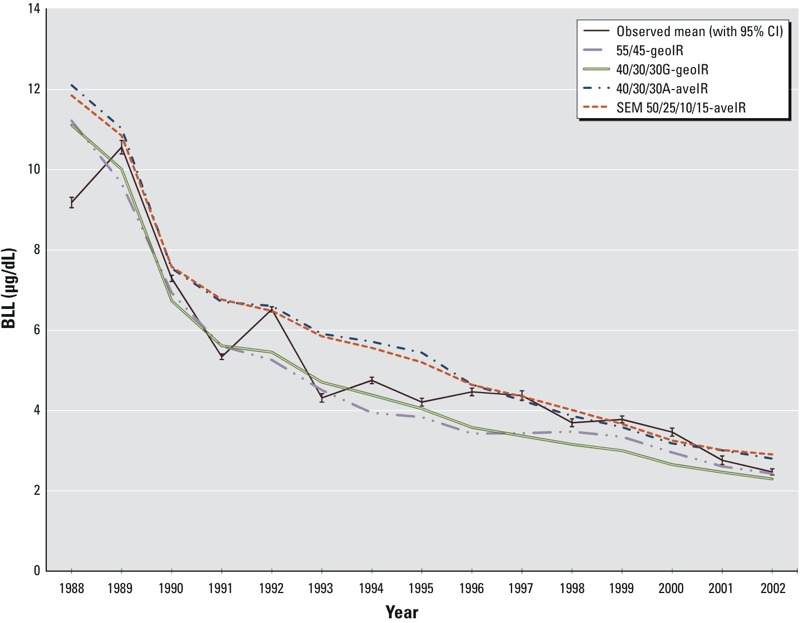
Observed and predicted geometric mean blood lead levels (BLLs) by year for four scenarios that best predict observed BLLs. Predicted geometric mean BLLs for the four scenarios are compared with observed BLLs from 1988 through 2002. Observed BLLs include error bars for the 95% confidence interval (CI). Abbreviations: aveIR, arithmetic mean ingestion rate; geoIR, geometric mean ingestion rate. 55/45 is the partition of dust/yard soil, 40/30/30 is the partition of dust/yard/community soil, and SEM 50/25/10/15 is the partition of dust/yard/neighborhood/community soil. Corresponding numeric data, with 95% CI and percentile distributions for each model and age, are provided in Table S1.

The four IR and partition scenarios with the best agreement are from the mid-range IRs shown in Figure 1 (i.e., 40/30/30G-geometric mean IR (geoIR), 55/45-geoIR, 50/25/10/15-arithmetic mean IR (aveIR), 40/30/30A-aveIR; the high- and low-range IRs, respectively, over- and underpredicted observed BLLs (data not shown). Figure 2 shows the results of the SSE and linear regression analyses for annual observed and predicted geometric mean BLLs for the four scenarios with the best agreement. Observed geometric mean BLLs ranged from > 8 μg/dL in the late 1980s to near 2 μg/dL in 2002, and observed geometric standard deviations (GSDs) ranged from 1.52 to 2.12 (*n* = 2,176). GSDs calculated from the IEUBK batch runs for these four scenarios ranged from 1.42 to 2.10, with medians around 1.7 (see Table S2), consistent with the IEUBK model default GSD of 1.6.

**Figure 3:**
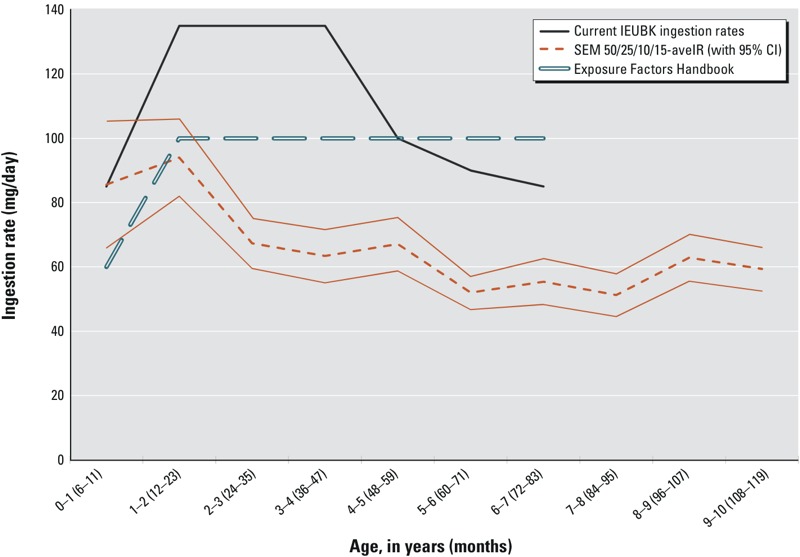
Mean age-specific ingestion rates (IRs) with 95% confidence intervals (CI) for the structural equations modeling (SEM) partition scenario. SEM 50/25/10/15 partition scenario (of dust/yard/neighborhood/community soil) with arithmetic mean IRs (aveIR) for ages 6 months–9 years, including 95% CI, are compared with current Integrated Exposure Uptake Biokinetic (IEUBK) model IRs and *Exposure Factors Handbook* IRs (ages 6 months–6 years only) (U.S. EPA 1994, 2011).

Each of the four scenarios represents a plausible source partition and estimated lead intake scenario, produces similar IR estimates (Table 5), and shows temporal variability in the SSE, with the largest SSEs in 1988 (see Table S3). The scenarios with the smallest total SSE for 1989–2002 were 40/30/30G-geoIR, 55/45-geoIR, and 50/25/10/15-aveIR. The 40/30/30A-geoIR was similar to the 50/25/10/15-aveIR and had the next smallest SSE for those same years. Although all four scenarios showed temporal variation in predicting observed BLLs, the 50/25/10/15-aveIR had the lowest SSEs in the early and later years of the cleanup (1989–1990 and 1996–2002, respectively), whereas the 40/30/30G-geoIR had the lowest SSE in the middle years of the cleanup (1991–1995). Additionally, linear regression indicated that the 50/25/10/15-aveIR and the 40/30/30A-aveIR scenarios were best-fit models due to a slope coefficient nearest 1.0, in combination with highest *r^2^*, largest *F*-statistic, and smallest sum of squared residuals (see Table S4). The age-specific IRs and 95% CIs for the 50/25/10/15-aveIR scenario are shown in Figure 3 because this scenario had the lowest SSEs in multiple years and was one of the best-fit linear regressions. Figure 3 also shows age-specific IRs recommended by U.S. EPA risk assessment guidance (U.S. EPA 1994, 2011).

**Table 5:** Mean age-specific soil/dust ingestion rates (mg/day) for four scenarios that best predict observed blood lead levels.

Age^*a*^ (years)	55/45^*b*^-geoIR	40/30/30G^*c*^-geoIR	40/30/30A^*c*^-aveIR	50/25/10/15^*d*^-aveIR	Average all models
0–1	92	82	76	86	84
1–2	100	89	90	94	93
2–3	72	64	66	67	67
3–4	65	58	62	63	62
4–5	69	62	63	67	65
5–6	54	49	50	52	51
6–7	54	49	54	55	53
7–8	51	47	50	51	50
8–9	57	53	61	63	59
9–10	58	54	57	59	57
Abbreviations: aveIR, arithmetic mean ingestion rate; geoIR, geometric mean ingestion rate. ^***a***^0–1 = 6–11 months, 1–2 = 12–23 months, 2–3 = 24–35 months, etc. ^***b***^Dust/yard soil. ^***c***^Dust/yard/community soil; G = geometric mean; A = arithmetic mean. ^***d***^Dust/yard/neighborhood/community soil.					

## Discussion

At the BHSS, children’s soil/dust exposures have been investigated since the 1970s, and the IEUBK model has been used to evaluate the dose–response relationship since 1986. Use of the IEUBK model default IRs, bioavailability and soil/dust partition failed to account for soil sources beyond the immediate home yard and consistently overpredicted observed BLLs. In 1990, the BHSS cleanup criteria were developed using the 40/30/30G partition accounting for community soils and reduced soil/dust lead uptake (compared with the IEUBK model default). The overprediction of BLLs using default IEUBK model values was resolved by lowering soil and dust lead bioavailability, although it could have been explained by several combinations of reduced IRs or bioavailability. However at the time, it was not possible to determine which was predominant. In this study, we used a newly available laboratory method to estimate soil and house dust ABS. The soil and house dust bioavailability results of 33% and 28%, respectively, are similar to the recommended 30% IEUBK model default values and those found in other BHSS studies (Maddaloni et al. 1998). These findings suggest that IRs, not ABS, should be reduced by about 40% from the IEUBK default values to best represent the dose–response relationship observed at the BHSS.

In this study, the more rigorous SEM pathways analyses resulted in several plausible models, all suggesting that community and neighborhood soil sources, in addition to the yard soil source, are independent contributors to total lead uptake and bioavailable lead in house dust. Others have recently confirmed the importance of soil beyond the immediate home yard (Laidlaw et al. 2014; Zahran et al. 2013a, 2013b). The 50/25/10/15-aveIRs were derived from the only partition including neighborhood soils and exhibited the lowest SSEs in multiple years. These IRs were calculated using arithmetic-mean neighborhood and community soil exposures. The central tendency statistic that better approximates geographic area exposures has been the subject of debate and remains unresolved; the arithmetic mean represents an aggregate biased by high or low concentrations, and the geometric mean is the most likely concentration in the prescribed area. Two of the four select models employed arithmetic means, one used the geometric mean, and the IEUBK model default scenario uses individual observations and assumes the effect of soils beyond the home yard is included in house dust. However, all four models produced similar IRs with the average nearly identical to the 50/25/10/15-aveIRs, indicating the source partition is critical in describing lead intake.

Age-specific and temporal effects, also examined with SEM, suggested children 6–23 months of age exhibited greater lead intake rates from house dust than older children, consistent with the study by Wilson et al. (2013). Additionally, SEM analyses including year-specific variables suggested dust intake rates for younger children may have been lower early in the cleanup (1989–1993) and higher during the middle years of the cleanup (1994–1998). However, only age- and year-specific intake rates of interior dust were statistically significant predictors (Table 3); consequently, age- and year-specific IRs for soil intake (yard, neighborhood, or community) were not included in our final model (data not shown). Several factors may have caused temporal variations in IRs, or partition coefficients. Aggressive LHIP education and intervention programs may have resulted in a temporary reduction in soil/dust intake by children. Alternatively, elevated dust loadings caused by flooding and construction activities may have exacerbated ingestion in the middle years of the cleanup. However, SEM and IEUBK model sensitivity analyses investigating alternate time period (years) variable constructs suggested that variation in calculated IRs may be an artifact of the source partitions, nature of the data, or progression of the cleanup. At the beginning of the cleanup, there was little difference between community soil and neighborhood soil concentrations. As area-wide cleanups predominated, these variable concentrations diverged between 1994 and 1998 and returned to similar concentrations by 2000 (TerraGraphics 2004). The 50/25/10/15 SEM is the only partition scenario that captures spatial differentiation in soil outside the home yard through the neighborhood soil variable. It is also possible that various periods of the cleanup exhibited different partition ratios from landscape changes or LHIP activities.

The truncated 1989–1998 database was used to derive the select SEM partition because from 1999 forward, the yard, neighborhood, and community soil variables were dominated by remediated homes. Lead concentrations were not measured in remediated yards. Instead, a nominal value of 100 mg/kg was assigned to represent the maximum allowable recontamination level. Replacement soils, and presumably yard soil concentrations immediately following remediation, averaged ≤ 50 mg/kg (LFR Inc. 2008; McCulley, Frick & Gilman Inc. 1997). Consequently, remediated soil lead concentrations were likely biased high and reflected less variation in the final years of the cleanup. Including 1999–2002 in the SEM analyses could bias the standardized coefficients for soil lead parameters used to estimate source effects.

Additionally, SEM coefficients were based on 1,571 of 4,019 observations in the 1989–1998 database. Most missing variable measurements for the SEM subset were house dust lead levels, implying the home lacked a vacuum cleaner, and were associated with likely dustier homes and higher BLLs (TerraGraphics 2004; U.S. EPA 2000; von Lindern et al. 2003a, 2003b). As a result of the missing house dust levels, mean values for key variables in the SEM subset differ from those in the parent database; particularly, mean absorbed lead was about 11% greater for children with no dust lead observation. Because total absorbed lead was allocated to source variables, higher absorbed blood lead implies potentially higher soil/dust IRs, absorption rates, or dust lead concentrations, or a combination thereof among these underrepresented children. The LHIP provides free loaner high-efficiency particulate arresting vacuum cleaners to residents to address this need.

This study is part of larger cleanup and public health response. It was not a designed experiment. The LHIP paid participants a modest fee for blood and house dust samples specifically to identify and provide follow-up services to children at risk. Factors such as self-selection, repeat blood leads, uncontrolled vacuum dust samples, lack of a home vacuum cleaner, intervention responses, other lead sources, community awareness, and assumed clean soil values could bias the IRs higher or lower. Many of these factors were discussed in detail by von Lindern et al. (2003b).

## Conclusions

The addition of *in vitro* soil and house dust bioavailability estimates to the BHSS lead health database facilitated analysis of absorbed and bioavailable soil/dust lead, which improves understanding of the dose–response relationship and supports improved estimates of total soil/dust IRs. Bioavailability was substantially underestimated in the original BHSS risk assessment. The IEUBK model, using default bioavailability and default soil/dust IRs, consistently overpredicted BLLs collected from > 50% of resident children, and this was likely attributable to overestimating IRs. Although remediation activities were based on an inaccurate combination of IRs and bioavailability estimates, remediation was nonetheless effective in achieving the objective of < 5% of children with BLLs ≥ 10 μg/dL.

Soil and dust IRs at the BHSS from 1988 through 2002 averaged 66 mg/day (95% CI: 57, 75 mg/day) for children 6 months–9 years of age, and peaked at 94 mg/day (95% CI: 82, 106 mg/day) at age 12–23 months. The estimated IRs were lower than both IEUBK default and the U.S. EPA *Exposure Factors Handbook* recommended values for all ages except the youngest age group (< 12 months) (U.S. EPA 2001, 2011). The average IRs are 40% less than IEUBK default recommendations and 30% lower than estimates in the *Exposure Factors Handbook* (shown in Figure 3), and are consistent with recent studies and reviews suggesting values < 100 mg/day (Bierkens et al. 2011; Moya and Phillips 2014; Ozkaynak et al. 2011; Wilson et al. 2013).

Soil/dust IRs are among the most sensitive variables in the IEUBK and other risk assessment models used at hazardous waste sites (Griffin et al. 1999; TRW Lead Committee 2014). Accurately estimating lead intake requires simultaneously quantifying both soil/dust IRs and the soil/dust source partition. Inclusion of neighborhood and community soil exposures is essential to estimating soil/dust lead intake. These findings suggest that approximately half of the lead intake is from house dust and half is from soil, equally attributed to the immediate home yard and surrounding neighborhood/community. Additionally, the importance of soil outside the home environment varies with distance, not property boundaries, and intake estimates should account for soil sources in the immediate neighborhood and greater community.

## Supplemental Material

(336 KB) PDF
