# Supplementary material for: Estimating Children’s Soil/Dust Ingestion Rates through Retrospective Analyses of Blood Lead Biomonitoring from the Bunker Hill Superfund Site in Idaho
Source: Environ Health Perspect. 2016 Jan 8;124(9):1462–70. doi: 10.1289/ehp.1510144 (PMC5010415; doi:10.1289/ehp.1510144)
Supplement: (336 KB) PDF [file ehp.1510144.s001.acco.pdf]

Environ Health Perspect
DOI: 10.1289/ehp.1510144

Note to readers with disabilities: *EHP* strives to ensure that all journal content is accessible to all readers. However, some figures and Supplemental Material published in *EHP* articles may not conform to 508 standards due to the complexity of the information being presented. If you need assistance accessing journal content, please contact ehp508@niehs.nih.gov. Our staff will work with you to assess and meet your accessibility needs within 3 working days.

Supplemental Material

Estimating Children's Soil/Dust Ingestion Rates through Retrospective Analyses of Blood Lead Biomonitoring from the Bunker Hill Superfund Site in Idaho

Ian von Lindern, Susan Spalinger, Marc L. Stifelman, Lindsay Wichers Stanek, and Casey Bartrem

Table of Contents

Table S1. Arithmetic mean ingestion rates (aveIR) and geometric mean ingestion rates (geoIR) with 95% confidence intervals (CI) and percentiles for the four partition scenarios

Table S2. Observed and predicted geometric mean blood lead levels (µg/dL) with geometric mean standard deviations (GSD) for four partition and ingestion rate scenarios, by year

Table S3. Sums of squared error (SSE) between observed and predicted blood leads for different partition and ingestion rate (IR) scenarios, by year

Table S4. Linear regression results comparing observed to Integrated Exposure Uptake Biokinetic model predicted blood lead means for different partition and ingestion rate (IR) scenarios (intercept forced through zero)

Table S1. Arithmetic mean ingestion rates (aveIR) and geometric mean ingestion rates (geoIR) with 95% confidence intervals (CI)[a] and percentiles for the four partition scenarios.

Partition	Age[b]	*n*	aveIR (95% CI)	geoIR (95% CI)	Percentiles						
					5	10	25	50	75	90	95
55/45[c]	0-1	60	132 (99,165)	92 (74, 116)	21	34	49	98	163	265	370
	1-2	190	154 (133,175)	100 (87,115)	17	24	56	106	209	331	493
	2-3	226	111 (96,127)	72 (64,82)	14	22	38	80	139	236	313
	3-4	225	102 (87,118)	65 (57,74)	13	18	32	67	135	219	305
	4-5	208	108 (93,122)	69 (60,79)	11	15	39	75	142	249	307
	5-6	226	82 (72,92)	54 (47,61)	10	16	29	63	107	171	224
	6-7	229	90 (75,104)	54 (47,61)	9	14	27	56	109	184	284
	7-8	239	77 (67,86)	51 (45,58)	9	13	27	53	107	169	233
	8-9	270	99 (87,111)	57 (50,66)	4	15	29	68	132	234	305
	9-10	255	93 (81,105)	58 (51,66)	8	18	32	63	111	201	303
40/30/30G[d]	0-1	60	108 (85,131)	82 (67,100)	22	34	46	89	138	210	298
	1-2	190	123 (109,137)	89 (79,101)	18	30	56	91	159	262	323
	2-3	226	90 (79,101)	64 (57,71)	14	22	37	66	113	190	229
	3-4	225	83 (73,92)	58 (52,66)	13	18	35	60	111	160	206
	4-5	208	87 (77,98)	62 (55,70)	12	19	37	66	118	178	240
	5-6	226	67 (61,74)	49 (43,55)	11	15	26	55	94	140	166
	6-7	229	74 (65,84)	49 (44,56)	9	15	26	56	93	149	217
	7-8	239	67 (59,75)	47 (42,53)	9	14	27	51	88	132	185
	8-9	270	84 (74,93)	53 (47,61)	3	19	30	61	110	185	231
	9-10	255	78 (69,87)	54 (49,61)	9	20	32	61	98	169	212

Partition	Age[b]	*n*	aveIR (95% CI)	geoIR (95% CI)	Percentiles 5	10	25	50	75	90	95
40/30/30A[d]	0-1	60	76 (60,92)	59 (48,71)	16	24	36	58	88	173	195
	1-2	190	90 (79,101)	65 (58,74)	16	23	40	67	110	196	229
	2-3	226	66 (58,73)	47 (42,53)	11	17	27	50	80	145	171
	3-4	225	62 (54,69)	43 (39,49)	9	13	26	46	79	123	160
	4-5	208	63 (56,71)	46 (41,52)	10	14	30	51	80	120	197
	5-6	226	50 (45,55)	36 (32,41)	9	11	20	38	73	103	128
	6-7	229	54 (47,60)	37 (33,42)	7	11	20	40	68	112	151
	7-8	239	50 (44,56)	35 (32,39)	7	12	19	38	66	98	129
	8-9	270	61 (55,68)	40 (35,45)	2	14	25	42	85	131	170
	9-10	255	57 (51,64)	41 (37,46)	7	17	25	43	79	119	159
50/25/10/15[e]	0-1	54	86 (66,105)	66 (54,80)	17	27	38	72	94	165	221
	1-2	174	94 (82,106)	69 (60,78)	16	22	42	69	123	188	250
	2-3	202	67 (59,75)	49 (43,55)	10	19	28	53	82	140	178
	3-4	209	63 (55,72)	45 (40,50)	10	14	26	47	76	130	156
	4-5	192	67 (59,75)	48 (43,55)	11	15	32	53	86	122	182
	5-6	208	52 (47,57)	38 (34,43)	10	12	23	41	74	102	126
	6-7	218	55 (48,62)	37 (33,42)	7	11	21	41	68	116	171
	7-8	228	51 (45,58)	36 (32,41)	7	12	21	41	68	105	120
	8-9	258	63 (56,70)	41 (36,47)	2	14	25	44	80	134	170
	9-10	245	59 (52,66)	42 (37,47)	8	17	25	43	80	116	171

aveIR=arithmetic mean ingestion rate; geoIR=geometric mean ingestion rate.

[a] (Upper CI,Lower CI)

[b] 0-1= 6-11 months; 1-2= 12-23 months, 2-3= 24-35 months, etc.

[c] dust/yard soil

[d] dust/yard soil/community soil; G=geometric mean; A=arithmetic mean

[e] dust/yard soil/neighborhood soil/community soil

Table S2. Observed and predicted geometric mean blood lead levels (µg/dL) with geometric mean standard deviations (GSD) for four partition and ingestion rate scenarios, by year.

Year	Observed		40/30/30G-geoIR		55/45-geoIR		50/25/10/15-aveIR		40/30/30A-aveIR	
	n	BLL (GSD)	*n*	BLL (GSD)	*n*	BLL (GSD)	*n*	BLL (GSD)	*n*	BLL (GSD)
1988	70	9.18 (1.74)	70	11.11 (1.48)	70	11.22 (1.63)	69	11.85 (1.47)	70	12.1 (1.42)
1989	49	10.56 (1.83)	49	10.01 (1.59)	49	9.66 (1.76)	48	10.85 (1.57)	49	11.03 (1.52)
1990	139	7.29 (1.61)	139	6.73 (1.82)	139	6.93 (1.98)	135	7.58 (1.78)	139	7.54 (1.78)
1991	162	5.34 (1.57)	162	5.61 (1.67)	162	5.60 (1.78)	157	6.76 (1.66)	162	6.71 (1.67)
1992	230	6.52 (1.65)	230	5.45 (1.74)	230	5.26 (2.00)	221	6.48 (1.68)	230	6.61 (1.68)
1993	199	4.31 (2.12)	199	4.71 (1.65)	199	4.51 (1.90)	195	5.85 (1.61)	199	5.91 (1.60)
1994	203	4.75 (1.79)	203	4.38 (1.67)	203	3.94 (1.92)	198	5.56 (1.64)	203	5.72 (1.64)
1995	153	4.21 (1.85)	153	4.04 (1.81)	153	3.84 (2.10)	148	5.2 (1.74)	153	5.44 (1.70)
1996	162	4.46 (1.84)	162	3.58 (1.73)	162	3.43 (1.97)	156	4.64 (1.69)	162	4.65 (1.69)
1997	100	4.37 (1.81)	100	3.36 (1.67)	100	3.43 (1.87)	95	4.35 (1.69)	100	4.25 (1.64)
1998	157	3.7 (1.88)	157	3.16 (1.64)	157	3.47 (1.80)	149	4.01 (1.62)	157	3.87 (1.58)
1999	179	3.78 (1.80)	179	3.00 (1.70)	179	3.35 (1.84)	158	3.66 (1.65)	179	3.58 (1.60)
2000	139	3.46 (1.84)	139	2.65 (1.65)	139	2.95 (1.80)	123	3.25 (1.61)	139	3.18 (1.55)
2001	117	2.76 (1.81)	117	2.46 (1.55)	117	2.61 (1.70)	88	3.01 (1.49)	117	3.01 (1.48)
2002	117	2.47 (1.52)	117	2.29 (1.58)	117	2.43 (1.74)	94	2.91 (1.59)	117	2.8 (1.51)
Minimum GSD		1.52		1.48		1.63		1.47		1.42
Maximum GSD		2.12		1.82		2.10		1.78		1.78
Median GSD		1.81		1.67		1.84		1.64		1.60

aveIR=arithmetic mean ingestion rate; geoIR=geometric mean ingestion rate.

BLL=blood lead level.

Table S3. Sums of squared error (SSE) between observed and predicted blood leads for different partition and ingestion rate (IR) scenarios, by year.

**Year**	**40/30/30G**			**55/45**			**SEM 50/25/10/15**			**40/30/30A**		
	n	**geoIR**	**aveIR**	***n***	**geoIR**	**aveIR**	***n***	**geoIR**	**aveIR**	***n***	**geoIR**	**aveIR**
1988	70	3.72	26.90	70	4.15	40.25	69	0.00	7.10	70	8.54	0.01
1989	49	0.30	6.20	49	0.82	8.62	48	5.08	0.07	49	0.22	4.68
1990	139	0.31	2.22	139	0.13	5.73	135	2.18	0.07	139	0.06	2.37
1991	162	0.07	4.09	162	0.07	6.50	157	0.04	1.91	162	1.86	0.06
1992	230	1.13	0.33	230	1.59	0.62	221	2.52	0.01	230	0.01	2.15
1993	199	0.15	3.24	199	0.04	3.76	195	0.04	2.33	199	2.55	0.04
1994	203	0.13	0.91	203	0.65	0.50	198	0.21	0.65	203	0.93	0.14
1995	153	0.03	1.06	153	0.14	1.16	148	0.04	0.97	153	1.52	0.00
1996	162	0.78	0.03	162	1.07	0.07	156	0.87	0.01	162	0.03	0.79
1997	100	1.02	0.00	100	0.89	0.12	95	1.05	0.01	100	0.02	1.17
1998	157	0.29	0.13	157	0.05	1.21	149	0.35	0.08	157	0.03	0.49
1999	179	0.61	0.00	179	0.19	0.69	158	0.62	0.00	179	0.04	0.96
2000	139	0.65	0.01	139	0.26	0.34	123	0.77	0.03	139	0.08	0.95
2001	117	0.09	0.13	117	0.02	0.60	88	0.09	0.10	117	0.06	0.15
2002	117	0.03	0.17	117	0.00	0.64	94	0.03	0.18	117	0.11	0.06
Sum (1988-02)	2,176	9.34	45.43	2,176	10.07	70.80	2,034	13.90	13.53	2,176	16.06	14.02
Sum (1989-02)	2,106	5.61	18.53	2,106	5.92	30.55	1,965	13.90	6.44	2,106	7.52	14.01
Sum (1989-90)	188	0.62	8.42	188	0.95	14.35	183	7.26	0.15	188	0.28	7.04
Sum (1991-95)	947	1.52	9.64	947	2.48	12.53	919	2.85	5.87	947	6.87	2.39
Sum (1996-02)	971	3.48	0.47	971	2.48	3.67	863	3.79	0.42	971	0.37	4.58

aveIR=arithmetic mean ingestion rate; geoIR=geometric mean ingestion rate.

Table S4. Linear regression results comparing observed to Integrated Exposure Uptake Biokinetic model predicted blood lead means for different partition and ingestion rate (IR) scenarios (intercept forced through zero).

Model	*n*	F-statistic	r^2	Slope Coefficient	Standard Error	Pr > |t|	Sum of Squared Residuals
40/30/30A-aveIR	15	2,792	0.995	1.062	0.020	<0.0001	0.219
40/30/30A-geoIR	15	1,864	0.993	0.904	0.021	<0.0001	0.237
55/45/-aveIR	15	3,366	0.996	1.149	0.020	<0.0001	0.212
55/45-geoIR	15	2,089	0.993	0.953	0.021	<0.0001	0.235
40/30/30G-aveIR	15	3,131	0.996	1.106	0.020	<0.0001	0.211
40/30/30G-geoIR	15	1,708	0.992	0.950	0.023	<0.0001	0.286
50/25/10/15-geoIR	15	2,441	0.994	0.907	0.018	<0.0001	0.183
50/25/10/15-aveIR	15	3,291	0.996	1.060	0.018	<0.0001	0.185

aveIR=arithmetic mean IR; geoIR=geometric mean IR; Pr=probability r^2=r-squared.

Criteria used to select the best fitting models: a slope coefficient nearest 1.0, in combination with highest r^2, largest F-statistic, and smallest sum of squared residuals
